# Digital Dentists: A Curriculum for the 21st Century

**DOI:** 10.2196/54153

**Published:** 2025-01-08

**Authors:** Michelle Mun, Samantha Byrne, Louise Shaw, Kayley Lyons

**Affiliations:** 1 Melbourne Dental School, Faculty of Medicine, Dentistry and Health Sciences, The University of Melbourne, 720 Swanston Street, Melbourne, 3000, Australia, 61 438986001; 2 Centre for Digital Transformation of Health, Faculty of Medicine, Dentistry and Health Sciences, The University of Melbourne, Melbourne, Australia

**Keywords:** digital health, digital transformation, informatics, ehealth, dentistry, dental informatics, curriculum, competence, capability, dental education

## Abstract

Future health professionals, including dentists, must critically engage with digital health technologies to enhance patient care. While digital health is increasingly being integrated into the curricula of health professions, its interpretation varies widely depending on the discipline, health care setting, and local factors. This viewpoint proposes a structured set of domains to guide the designing of a digital health curriculum tailored to the unique needs of dentistry in Australia. The paper aims to share a premise for curriculum development that aligns with the current evidence and the national digital health strategy, serving as a foundation for further discussion and implementation in dental programs.

## Introduction

As the world continues to be digitally transformed, there are increasing expectations for health care providers to use technology and handle health information safely and ethically [1]. It is likely that future health professionals will also need to think critically about how digital health technologies can be used to transform models of care [2].

Digital health and informatics remain relatively new curriculum topics for many health professions, including dentistry. Defining the relevant curricular objectives in entry-to-practice degrees can be particularly challenging for several reasons. First, there are several definitions and conceptualizations of the term “digital health” [1,3-6]. Second, the implementation of digital health education in health profession degrees has largely been ad hoc, with different schools adopting varied approaches [7]. This has resulted in inconsistent learning outcomes and a fragmented understanding of digital health competencies for health profession graduates. Third, although a multitude of digital health competency frameworks exist [8-11], there is a notable absence of shared curriculum models specific to dentistry. Therefore, dentistry educators are not aware of or struggle to adopt best practices in the teaching of digital health.

In this viewpoint, we argue that there is a need to integrate digital health education into dentistry curricula to prepare future practitioners for the increasingly digitized health care environment. Specifically, we propose a distinct point of view for defining "digital health" in dental education, a structured set of domains to guide the design of digital health curriculum, and a framework for curriculum development that aligns with current evidence.

## Beginning With the End in Mind

In Australia, a country that ranks consistently high for digital health maturity [12], clear digital health objectives are set out through national strategies such as the Australian Digital Health Agency (ADHA) Capability Action Plan (2023) and the National Healthcare Interoperability Plan (2023‐2028) [1,13]. The government body for digital health (ie, the ADHA), peak bodies such as the Australasian Institute of Digital Health (AIDH), and digital health innovation centers all identify building workforce capability as a critical part of achieving digital transformation of health care [1,13-15]. These organizations envision a future where health professionals will work in integrated and multidisciplinary environments. Digital health education in entry-to-practice degrees is thus a core element of advancing workforce capability; however, the specific content, including priority areas of knowledge and skills, must be tailored to the unique demands of each discipline and local context. For dentistry in Australia, this means aligning the curriculum with the national digital health strategy while addressing the current maturity level and future needs of the dental profession.

## Reframing “Digital Health” for the Next Generation of Australian Dental Practitioners

A lack of standardized digital health education in entry-to-practice degrees in Australia has been recognized for decades [16-18] and has been demonstrated by gaps in workforce competency [19]. Global interest in the digital transformation of health care is accelerating, catalyzed by the COVID-19 pandemic and advances in artificial intelligence (AI). However, not all health professions have advanced equally considering their digital transformation. In Australia, as in many countries, the dental sector remains traditionally siloed from the rest of the health care system and faces fragmentation in its information systems [20]. In the dental sector, the most progress in digital health has been observed in restorative and surgical procedures, where technology is directly integrated into clinical workflows [21]. For example, conventional manual techniques and laboratory workflows for the design and fabrication of dental restorations have evolved into in-house, fully digitized workflows with the application of intraoral scanners and chairside milling machines [22]. In contrast, dentistry has a relatively nascent data culture [23], with less focus on the broader scope of digital health, which we define in this viewpoint to encompass virtual care, remote monitoring, mobile health (mHealth), wearables, big data analytics, platforms, and “the exchange of data and sharing of relevant information across the health ecosystem creating a continuum of care” [4].

Developing digital health–capable dentists thus involves more than simply teaching the technical aspects of digital tools used in service delivery; it requires a shift towards understanding how digital data can inform clinical decisions, enhance patient care, and contribute to system-wide improvements. This conceptual change is crucial for moving from a service-focused practice to one that leverages digital health as an integral part of modern dental care. The Learning Health Systems (LHS) framework [24] is one example of how to help dental professionals characterize digital health. LHS are health care environments where science, informatics, incentives, and culture align to promote continuous improvement and innovation. In these systems, best practices are embedded in care, patients actively participate, and new knowledge is generated from every care experience [25]. Building on this vision, dental education should emphasize models where digital health is central to both practice and continuous improvement. This approach will foster digital health capability by cultivating a deeper understanding of how and why digital health technologies enable the delivery of high-quality, safe, and sustainable care.

Textbox 1 outlines the questions that can guide the development of a digital health curriculum for entry-to-practice dental education. These questions are intended to help educators and curriculum developers define clear goals aligned with the specific needs of the discipline and the local health care context.

Textbox 1. **Defining the goals of digital health curriculum for entry-to-practice degrees.**

- What is outlined in national and local digital health strategies for the next 5-10 years? What does the political and funding environment look like?
- What are the digital health-related accreditation standards of the profession?
- What does the current and future digital health maturity of the primary work environments of your graduates look like?
  - Consider the difference in goals for:
    - A rural school where graduates may work in areas with limited digital maturity
    - A health discipline or specialty where graduates will typically work in tertiary care rather than primary care

## Considerations for Curriculum Development

The Australian Dental Council (ADC) recently revised its competencies for newly qualified dental practitioners; they updated the requirement to include “using digital technologies and informatics to manage health information and inform person-centred care” [26]. This prompted the authors to develop a digital health curriculum to be implemented in a higher education institution that has graduating dental professionals in Australia. As per the best practice in curriculum development [27], we considered the existing digital health competency and capability frameworks as part of our curricular needs assessment. An environmental literature scan found that only a few frameworks had been created specifically for dentistry or involved dental experts in their consultations, reflecting a lag in dentistry’s digital health participation (Multimedia Appendix 1). As a result, not all topics in these existing frameworks were relevant or current to the reality of training dental professionals in Australia, who will predominantly work in small clinics in primary care, in practices with varying digital health maturity [28,29]. An exception was the digital dentistry curriculum proposed by the American College of Prosthodontists [30], which is well-researched but focused solely on digital skills for prosthodontics. This highlighted a gap in resources to support the broader skill set of graduating dentists in Australia, as outlined by the ADC.

The process of designing higher education courses aims to align industry standards with a scaffolded approach for developing effective learning outcomes that produce work-ready graduates. While the ADC’s revised competency served as a catalyst for curriculum development, our efforts extended beyond the ADC’s scope to meet standards such as those overseen by the Tertiary Education Quality and Standards Agency (TEQSA), which performs the quality assurance checks for all participants, delivered as part of higher education in Australia. TEQSA’s emphasis on authenticity in curricula design, as well as contemporary leading practice [31,32], influenced our approach towards designing a curriculum that not only meets regulatory competencies but also prepares students for practical, professional challenges in the evolving digital health landscape.

Finally, a critical component of our approach was to tailor the curriculum to the local context. While internationally recognized informatics competencies [33] often underpin digital health capability frameworks, they do not alone fully capture the breadth and nuances of digital health proficiency. Digital health encompasses a range of skills, including digitally enabled clinical processes, care pathways, and behavior change management, all of which are shaped by local variations in digital health maturity and sociocultural contexts. Furthermore, curriculum development often occurs under significant time and resource constraints, requiring an approach that is rigorous but targeted. For example, rural schools may not yet prioritize AI competencies if electronic health records are not yet in use locally.

## Key Domains

Two frameworks were selected to inform the development of the dental digital health curriculum, both of which are government-sponsored, peer-reviewed, and directly relevant to the Australian setting [Textbox 2]. The domains in Table 1 are an abridged synthesis created by the authors, drawing on elements from the two selected frameworks. This reimagined structure is intended to facilitate the development of a digital health curriculum for dentistry, aligning learning objectives, instruction, and assessment with the national strategy in Australia.

Textbox 2. **Frameworks selected to inform development of the dental digital health curriculum.**

1. Framework 1 (2018): eHealth Capabilities Framework for Graduates and Health Professionals [34]. This framework was developed by the University of Sydney and eHealth New South Wales, consisting of a tri-phase literature review, focus groups with faculty and government representatives (n=23), and a Delphi method refinement with 4 iterations. The framework is structured in 4 domains and describes recommended knowledge and skills for health professions graduates in digital health.
2. Framework 2 (2021): Digital Health Capability Framework for Allied Health Professionals [35]. This framework was developed by the Department of Health, Victoria, and consisted of a 3-part development program including a competency framework review, expert discussion panel interviews (n=28), and an online survey of Victorian allied health professionals (n=164). This document draws from Framework 1 and is similarly structured into 4 domains of 3-6 subdomains, with the addition of levels of digital health proficiency ranging from Foundation, Consolidation, Expert, and Leadership.

**Table 1.** Domains and goals for digital health curriculum for an entry-to-practice dental degree.

Domain	Learning goal	Suggested learning topics
Digital transformation of health	Newly graduated dental practitioners will actively lead the digital transformation of dentistry by using technology to deliver patient-centred care and by recognizing the role of data and analytics in improving it.	Electronic health records, digital dentistry (radiography, intraoral scanning, CAD/CAM^a^, and other digital workflows) data, interoperability and learning health systems, artificial intelligence
Legislation, policy, and governance	Newly graduated dental practitioners will drive improvements in the privacy and security of patient data, and model the safe, ethical, and responsible use of digital health technologies in the dental practice.	Data privacy and cybersecurity
Digital health for patients	Newly graduated dental practitioners will promote patient engagement in health care, prescribe appropriate digital resources, and support digital health literacy.	Digital health literacy, patient engagement in health care, and digital health equity
Digital professionalism	Newly graduated dental practitioners will model a professional and appropriate digital identity.	Social media and digital professionalism

a CAD/CAM: computer-aided design/computer-aided manufacturing.

The first domain recognizes that along with technical proficiency in digital clinical workflows, dental practitioners must be able to think in multidisciplinary terms of the flow of data and information across health care [13]. Dental practitioners must understand the importance of informatics, interoperability, and a quality improvement mindset to be the building blocks for creating LHS [24,25].

The second domain recognizes the role of the dental practitioner in safe and ethical governance of patient data across digital workflows, noting that health care is the consistently top-reporting sector for data breaches in Australia [36].

The third domain recognizes the shift from the paternalistic model of health care towards a person-centered one where the person receiving care plays an active role in shared health care decision-making. The OpenNotes mandate in the US is a good example of this [37]. This domain is also particularly relevant to the rapid pace of AI development and the accessibility of generative AI models that patients may use to access health (mis)information. Dental practitioners must understand digital health literacy; how patients may engage with digital health technologies and services; and the uses, ethics, benefits, and risks of AI in health care.

The fourth domain recognizes that dental practitioners must develop a professional identity, which is multidimensional across social media and the internet. The obligation for a dental practitioner to uphold their professional code of conduct is binding for both their in-person and digital profiles [38].

This holistic overview of digital health in dentistry is a step towards addressing the observation that digital health education tends to be focused on medical degrees—mostly in electives or single-unit areas such as telehealth—and in utilizing diverse approaches for delivery, development, and assessment [39]. A similar observation was found during our curricular needs assessment, revealing a strong focus in single content areas such as telehealth and digital dentistry, but confirming opportunities to facilitate a more coordinated and comprehensive learning pathway to support full digital health competency.

## Final Thoughts

Viewing dentistry through a “digital health” lens may seem like a small matter. However, the change in perspective for dental educators is important. Dentistry has traditionally focused on individual patient care and procedural intervention, but contemporary health care is increasingly shaped by system-level forces. AI, interoperability, value-based care, and increasing consumer participation are now current realities [40-43]. The potential for digital health to drive meaningful systemic improvements in oral health and health care cannot be truly realized without first building the necessary capability at the graduate level. Consequently, these topics can and should be taught in a structured manner in entry-to-practice dental education.

Critically, although newer generations are often seen as digitally adept, they do not automatically master the necessary digital skills simply from being exposed to technology [44]. This gap in digital competency underscores the importance of intentional curriculum design. Universities are increasingly using the approach of constructive alignment to enhance outcome-based education [45], and this approach should be used to design a longitudinal digital health curriculum that can align with the intended graduate attributes.

This viewpoint has outlined the premise for designing a digital health curriculum in dentistry, using a structured set of domains based on current evidence and adapted to the Australian context. The proposed domains provide a foundation for educators to build a curriculum that aligns with the unique needs of dental professionals and the national strategy for digital health. This approach is intended for integration into the University of Melbourne’s dentistry program and aims to encourage the further development and discussion of digital health education within dental programs, both nationally and globally.

## Conclusion

It can be difficult for educators to define digital health curriculum that is both evidence-based and relevant to their discipline and local context; to design it is to predict the future. However, keeping pace involves changing our view of digital health in dentistry. A common understanding about the language of digital health is important for developing health professionals who will be able to navigate the environment of the modern health care system. We found that existing digital health capability frameworks were useful to define a view of digital health across an entry-to-practice dental degree, and high level roadmaps and frameworks are valuable to envision a future-ready dental graduate who can embrace the next wave of digital transformation. This perspective will be useful for developing the curriculum aligned with the national vision of building workforce capability and realizing the aim of safe, connected care.

## Supplementary material

10.2196/54153 Multimedia Appendix 1 Digital health capability and competency frameworks considered for curriculum development in dentistry.

## Abbreviations

ADC: Australian Dental Council
ADHA: Australian Digital Health Agency
AI: artificial intelligence
AIDH: Australasian Institute of Digital Health
CAD/CAM: computer-aided design/computer-aided manufacturing
LHS: learning health system
mHealth: mobile health
TEQSA: Tertiary Education Quality and Standards Agency
