# Supplementary material for: Digital Dentists: A Curriculum for the 21st Century
Source: JMIR Med Educ. 2025 Jan 8;11:e54153. doi: 10.2196/54153 (PMC11735848; doi:10.2196/54153)
Supplement: Multimedia Appendix 1 [file mededu-v11-e54153-s001.docx]

Appendix 1. Digital health capability and competency frameworks considered for curriculum development in dentistry.

| Author/Organisation | Country | Specific to dentistry? | Specific to digital health or health informatics? | Relevant for entry-to-practice health professions degree? | Holistic (not sub-component of digital health)? | Discipline-specific? | Dentistry included in consultation? |
| --- | --- | --- | --- | --- | --- | --- | --- |
|  |  |  |  |  |  |  |  |
| American College of Prosthodontists, 2018 [30] | USA | ✓ | ✓ | ✓ | - | ✓ | ✓ |
| Brunner et al., 2018 [34] | Australia | - | ✓ | ✓ | ✓ | - | ✓ |
| University of Adelaide, 2018 [46] | Australia | - | - | ✓ | - | - | ? |
| Jisc, 2015 [47] | UK | - | - | ✓ | - | - | ? |
| Academy of Medical Royal Colleges, 2011 [48] | UK | - | ✓ | ✓ | ✓ | - | ✓ |
| Canada Health Infoway, 2014 [49] | Canada | - | ✓ | ✓ | ✓ | - | - |
| HITCOMP, 2020 [50] | Global | - | ✓ | ✓ | - | - | ✓ |
| Jidkov et al, 2019 [51] | UK | - | ✓ | - | ✓ | ✓ | - |
| Thye et al, 2018 [52] | Global | - | ✓ | ✓ | ✓ | - | ? |
| Pontefract & Wilson, 2019 [53] | UK | - | ✓ | ✓ | - | - | - |
| Khurana et al, 2022 [54] | USA | - | ✓ | ✓ | ✓ | ✓ | - |
| Littlewood et al., 2021 [35] | Australia | - | ✓ | ✓ | ✓ | - | - |
| AMC, 2022 [55] | Australia | - | ✓ | ✓ | ✓ | ✓ | - |
| NHS, 2017 [56] | UK | - | - | ✓ | - | - | ? |
| ADHA, 2020 [57] | Australia | - | ✓ | - | ✓ | ✓ | - |
| ADHA, 2020 [58] | Australia | - | ✓ | ✓ | ✓ | - | ✓ - not major stakeholder; one representative in 1/9 workshops |
| ADHA, 2022 [1] | Australia | - | ✓ | ✓ | ✓ | - | ✓ |
| Röhrig et al, 2013 [59] | Germany | - | ✓ | ✓ | ✓ | ✓ | - |
| Hübner et al., 2020 [60] | Global | - | ✓ | - | ✓ | ✓ | - |
| Valenta et al., 2018 [33] | Global | - | ✓ | - | ✓ | ✓ | ? |
| Khairat & Feldman, 2020 [61] | USA | - | - | ✓ | ✓ | - | ? |

✓ Yes; - No; ? Unclear/NA
